# Genome Sequence of *Rheinheimera salexigens* sp. nov. Isolated from a Fishing Hook off O‘ahu, Hawai‘i

**DOI:** 10.1128/genomeA.01390-16

**Published:** 2016-12-15

**Authors:** Xuehua Wan, Shaobin Hou, Kazukuni Hayashi, James Anderson, Stuart P. Donachie

**Affiliations:** a Advanced Studies in Genomics, Proteomics and Bioinformatics, University of Hawai‘i at Mānoa, Honolulu, Hawai‘i, USA; b Department of Microbiology, University of Hawai‘i at Mānoa, Honolulu, Hawai‘i, USA; c The Hawai’i Institute of Marine Biology, Kane’ohe, Hawai‘i, USA; d Department of Biology, University of Hawai‘i at Mānoa, Honolulu, Hawai‘i, USA

## Abstract

*Rheinheimera salexigens* KH87^T^ is an obligately halophilic gammaproteobacterium. The strain’s draft genome sequence, generated by the Roche 454 GS FLX+ platform, comprises two scaffolds of ~3.4 Mbp and ~3 kbp, with 3,030 protein-coding sequences and 58 tRNA coding regions. The G+C content is 42 mol%.

## GENOME ANNOUNCEMENT

Type strains of species in the genus *Rheinheimera* have been cultivated from a range of aquatic and terrestrial environments (1–3). Beyond formal descriptions, in which growth parameters are usually defined for a species, little information is available on specific roles of *Rheinheimera* species in the environment (4, 5). In this respect, we cultivated strain KH87^T^ from a circle fishing hook that had been baited and suspended in seawater north of O‘ahu, HI, as part of an investigation of bacteria found in the mouths of 11 shark species in the Pacific Ocean.

Genomic DNA was isolated from KH87^T^ cells pelleted from a ~48-h culture in ZoBell’s 2216A Marine broth, using lysozyme and proteinase K lysis, with an additional cetyltrimethylammonium bromide (CTAB) incubation step, followed by phenol extraction and isopropanol precipitation. A total of 33.7 Mb of shotgun reads and 57.4 Mb of 8-kb paired-end reads were generated in the Roche 454 GS FLX+ platform. Newbler 2.8 assembled four scaffolds containing 3,445,563 bp (scaffold *N*_50_, 3,437,899 bp). Most gaps were closed *in silico* (6). Upon closing gaps totaling 15.3 kbp, the draft genome contained two scaffolds, spanning 3,438,923 bp and 2,958 bp. The genome’s G+C content is 42%.

Genome annotation was performed in the NCBI Prokaryotic Genome Annotation Pipeline (PGAP) and the Rapid Annotation Using Subsystem Technology (RAST) server (7–9). PGAP identified 3,030 protein-coding genes, 58 tRNA-coding regions, and 75 pseudogenes. RAST identified 3,196 protein-coding genes, 58 tRNA-coding regions, and 463 function-related subsystems. Genes for flagella and chemotaxis are present. RAST also predicted five phage component proteins and one integron. The biofilm matrix-producing operon *pelA*-*pelG* was predicted by RAST. In BLASTP, we manually predicted homologs of RpfF, RpfC, and RpfG, proteins that participate in virulence and cell-cell communication through production and sensing of a diffusible signal factor (DSF) in plant-pathogenic *Xanthomonas* species (*E* value <1e^-10^) (10, 11). Further bioinformatics analysis and wet-lab experiments will help verify and characterize the potential DSF signaling pathway to extend our knowledge of marine bacteria cell communication, signal transduction, and biofilm formation.

### Accession number(s).

This whole-genome shotgun project has been deposited in DDBJ/EMBL/GenBank under accession number MKEK00000000. The version described in this paper is the first version, MKEK01000000. The 16S ribosomal gene sequence has been deposited at GenBank under accession number KP026120.
